# A Polymorphism in the Processing Body Component Ge-1 Controls Resistance to a Naturally Occurring Rhabdovirus in *Drosophila*

**DOI:** 10.1371/journal.ppat.1005387

**Published:** 2016-01-22

**Authors:** Chuan Cao, Michael M. Magwire, Florian Bayer, Francis M. Jiggins

**Affiliations:** Department of Genetics, University of Cambridge, Cambridge, United Kingdom; Stanford University, UNITED STATES

## Abstract

Hosts encounter an ever-changing array of pathogens, so there is continual selection for novel ways to resist infection. A powerful way to understand how hosts evolve resistance is to identify the genes that cause variation in susceptibility to infection. Using high-resolution genetic mapping we have identified a naturally occurring polymorphism in a gene called *Ge-1* that makes *Drosophila melanogaster* highly resistant to its natural pathogen *Drosophila melanogaster* sigma virus (DMelSV). By modifying the sequence of the gene in transgenic flies, we identified a 26 amino acid deletion in the serine-rich linker region of Ge-1 that is causing the resistance. Knocking down the expression of the susceptible allele leads to a decrease in viral titre in infected flies, indicating that *Ge-1* is an existing restriction factor whose antiviral effects have been increased by the deletion. Ge-1 plays a central role in RNA degradation and the formation of processing bodies (P bodies). A key effector in antiviral immunity, the RNAi induced silencing complex (RISC), localises to P bodies, but we found that *Ge-1*-based resistance is not dependent on the small interfering RNA (siRNA) pathway. However, we found that Decapping protein 1 (DCP1) protects flies against sigma virus. This protein interacts with Ge-1 and commits mRNA for degradation by removing the 5’ cap, suggesting that resistance may rely on this RNA degradation pathway. The serine-rich linker domain of Ge-1 has experienced strong selection during the evolution of *Drosophila*, suggesting that this gene may be under long-term selection by viruses. These findings demonstrate that studying naturally occurring polymorphisms that increase resistance to infections enables us to identify novel forms of antiviral defence, and support a pattern of major effect polymorphisms controlling resistance to viruses in *Drosophila*.

## Author Summary

Hosts and their pathogens are engaged in a never-ending arms race, and hosts must continually evolve new defences to protect themselves from infection. In the fruit fly *Drosophila melanogaster* we show that virus resistance can evolve through a single mutation. In flies that are highly resistant to a naturally occurring virus called sigma virus we identified a deletion in the protein-coding region of a gene called *Ge-1*. We experimentally confirmed that this was the cause of resistance by deleting this region in transgenic flies. Furthermore, we show that even the susceptible allele of Ge-1 helps protect flies against the virus, suggesting that this mutation has made an existing antiviral defence more effective. Ge-1 plays a central role in RNA degradation in regions of the cytoplasm called P bodies, and our results suggest that this pathway has been recruited during evolution to protect *D*. *melanogaster* against sigma virus. The protein domain that contains the deletion has experienced strong selection during its evolution, suggesting that it may be involved in an ongoing arms race with viruses.

## Introduction

Hosts and their pathogens are engaged in a never-ending arms race, where the evolution of new defences in turn selects for pathogens that can overcome those defences. In order to understand how hosts are evolving resistance, it is necessary to investigate the genes that cause variation in the susceptibility to infection in the wild. Resistance can evolve not only by altering the host immune defences, but also by changing host factors that are hijacked by the pathogen for its own benefit. For example, bacteria commonly evolve resistance to phages by modifying surface receptors needed to bind and enter cells [1]. Therefore studying natural variation can identify novel forms of host defence that are not apparent from classical immunology.

Viruses are important pathogens of insects, but the antiviral defences of insects are still comparatively poorly understood. The most important immune defence against RNA viruses is RNAi, whereby double-stranded viral RNA is cleaved into short RNAs by Dicer 2, and these then guide the degradation of viral RNA by Argonaut 2 [2]. In response, many insect viruses have evolved Viral Suppressors of RNAi (VSRs) that block RNAi in a variety of different ways [2–5]. Several other pathways and the endosymbiotic bacterium *Wolbachia* have been implicated in the antiviral immunity in *Drosophila* [6–16], but these are mostly relatively poorly understood.

In insect populations there is extensive genetic variation in susceptibility to viral infection. In *Drosophila* genome-wide association studies have shown that much of this genetic variation can be explained by a small number of major effect polymorphisms [17,18]. Interestingly, these major effect polymorphisms were only seen when natural coevolved viral pathogens of *Drosophila* were used (*Drosophila melanogaster* sigma virus (DMelSV) and Drosophila C virus (DCV), but not when flies were infected with viruses from other species. Haplotypes carrying the resistant alleles of two genes–*ref(2)P* and *CHKov1* –carry very little genetic variation, indicating that they have been driven to a high frequency by natural selection [17,19–21]. It has been estimated that *ref(2)P* has experienced a selective sweep within the last 1000–7000 years, while *CHKov1* swept to a higher frequency within the last few hundred years (although the allele may be much older) [20,22,23]. Therefore, it appears that selection for resistance to the sigma virus is driving major-effect resistance alleles through populations, and the genetic variation observed in nature is the result of these transient polymorphisms.

In *Drosophila melanogaster* three of these major-effect polymorphic resistance genes have been identified at the molecular level, although their mode of action remains uncertain. The first of the genes to be cloned is known as *ref(2)P* or *p62* and confers resistance to sigma virus. Resistance arose through a mutation from a Gln-Asn to a single Gly in the PB1 domain (a protein interaction module) of the protein [19,21,22,24]. P62 is an adaptor protein which, among other functions, selectively targets polyubiquitinated substrates for degradation by autophagy. In mammals it targets bacteria for degradation by autophagy [25] [26], and autophagy is known to protect flies from infection of vesicular stomatitis virus (VSV), a relative of sigma viruses [11]. Therefore *p62* may contribute to sigma virus defence through its role in autophagy. The second gene to have been identified was *CHKov1* [17]. Three alleles of *CHKov1* genes occur in natural populations of *D*. *melanogaster*, each conferring a different level of protection to DMelSV infection. The ancestral allele has the most susceptible phenotype, and a transposable element (*Doc1420*) insertion into the protein coding sequence of *CHKov1* has dramatically increased flies’ resistance to DMelSV. The insertion results in truncation of mRNA, leading to four different transcripts [20]. This allele is the most common allele in North American populations, being found at a frequency of 0.82 in North Carolina. The third allele is the most resistant and is the result of two duplications, with rearrangement of three copies of both the truncated *CHKov1* allele and *CHKov2* (one of which is truncated) [17]. The mechanism by which *CHKov* genes confer protection is unclear. The third gene to have been identified is *pastrel*, which provides resistance to DCV [18,27].

DMelSV infects up to 18% of flies in natural populations [28–30]. It is a single-stranded, negative-sense RNA virus from the Rhabdovirus family. It is only transmitted vertically from parent to offspring, and is therefore a host-specific pathogen of *D*. *melanogaster*. Compared to some viruses that cause high levels of mortality, infection by sigma virus appears relatively benign. However it can reduce egg viability and infected adults may be less likely to survive to overwinter than uninfected ones [31]. Both field and laboratory studies have estimated that sigma virus infected flies suffer a reduction in fitness of approximately 20–30% [32,33]. Therefore as sigma viruses are common and costly parasites of *D*. *melanogaster*, there is selection for flies to evolve resistance.

Aside from the two polymorphic genes known to affect sigma virus–*P62* and *CHKov1* –three other naturally polymorphic genes that alter the replication or transmission of sigma virus have been mapped to fairly large regions of the *Drosophila* genome: *ref(1)H*, *ref(2)M* and *ref(3)O[34]*. Among them *ref(2)M*, a naturally polymorphic resistance gene, was mapped to a region of the left arm of the second chromosome between two visible markers in 1978[34]. There is little study of this gene since. Here we combine linkage mapping, association studies and reverse genetics to map this gene and identify the polymorphism causing resistance.

## Results

### High resolution genetic mapping locates a major-effect polymorphism controlling resistance to DMelSV

**Fig 1:**
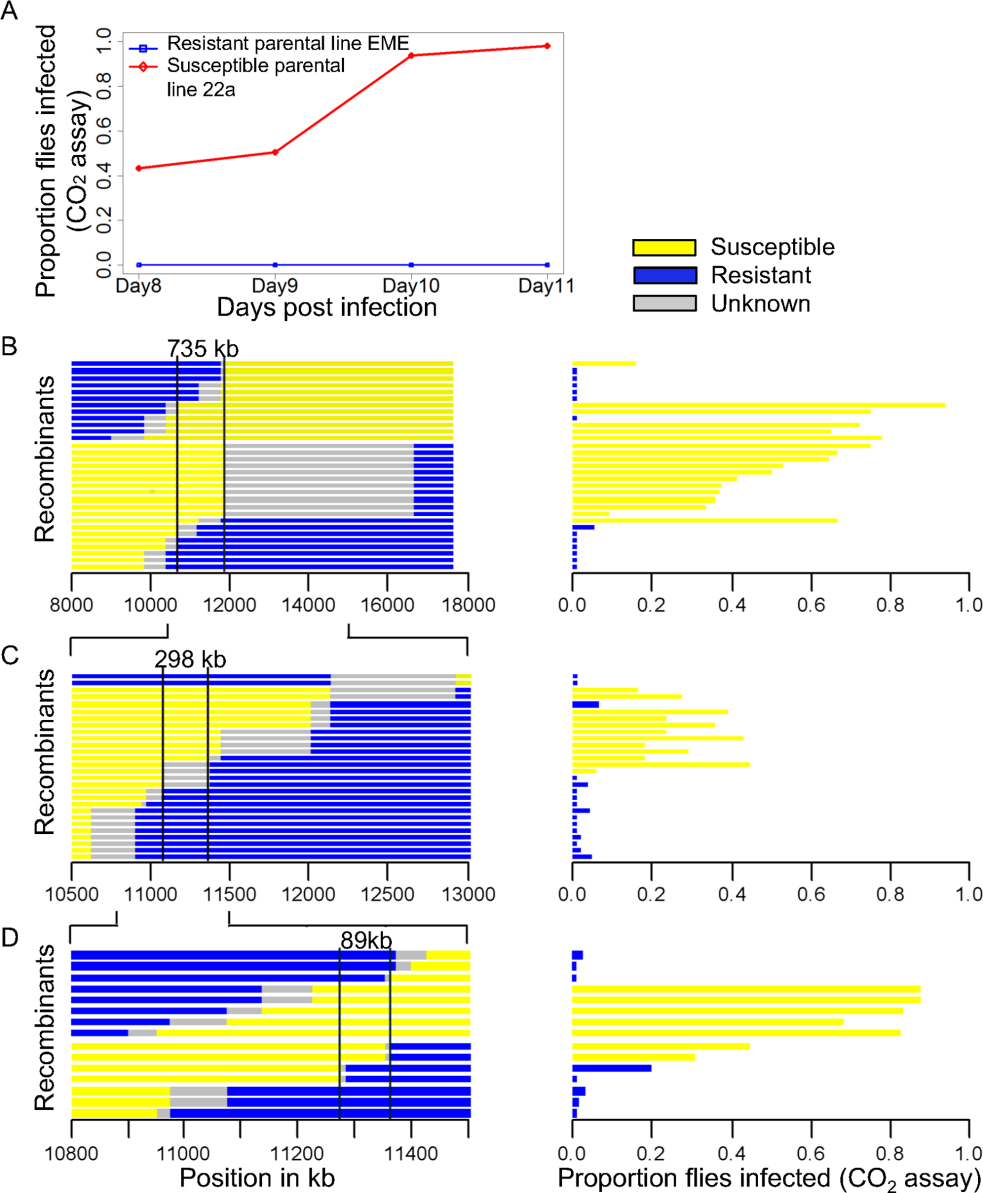
Linkage map of the gene controlling susceptibility. (A) The susceptibility of the resistant (EME) and the susceptible (22a) parental lines was measured by injecting the flies with the DMelSV and testing whether they become paralysed after exposure to CO2 (a symptom of infection). Panel B, C and D are the three successive experiments where recombinants in the region known to contain the gene were selected using molecular markers and made homozygous. On the left are the genotypes of the recombinants, with chromosomal regions from the resistant parent in blue and the susceptible parent in yellow, and the location on chromosome 2 in the *D*. *melanogaster* genome R5. The infection rate is shown on the right, with lines classed as resistant in blue and susceptible in yellow. There is a near-perfect association between genotype and phenotype in the regions between the vertical lines. Experiment (B) narrowed down the region to 735 kb using 31 recombinant lines. (C) Further narrowed down the region to 298 kb using 28 recombinant lines. (D) Narrowed down the region to 89 kb using 15 recombinant lines.

The susceptibility of *D*. *melanogaster* to DMelSV is affected by several naturally polymorphic genes that have been roughly mapped in the genome but have not been identified at the molecular level [34,35]. One of these genes, which has previously been called *ref(2)M*, has been approximately mapped to a region of chromosome 2 [34]. Starting with two fly stocks known to carry different alleles of this gene: EME (resistant) [34] and 22a (susceptible) [17], we found that its effect on the DMelSV was dramatic—10 days after injecting the virus, 94% of flies carrying the susceptible allele showed symptoms of infection after exposure to CO_2_ compared to only 3% of flies with the resistant allele (Fig 1A).

To map the location of this gene to a smaller region, we crossed the resistant and susceptible fly lines, and then used balancer chromosomes to generate stocks that carried a homozygous recombinant 2^nd^ chromosome. As we knew the approximate location of the gene, we only retained 31 lines that had recombined between two molecular markers at 36 and 52 cM on the left arm of chromosome 2. Further molecular markers were then scored in this region, and these lines were then injected with DMelSV and tested for infection using the CO_2_ assay. These recombinant lines had a bimodal distribution of infection rates, with most lines having infection rates of either 0% or greater than 40%. There was a region of approximately 735kb where there was a perfect correspondence between the genotype of the fly and infection rate (Fig 1B; Wilcoxon Rank Sum Test: *W* = 225.5, *P* = 8.4x10^-6^).

To further refine the location of the gene, we repeated this experiment to generate informative recombinants in the candidate region. To reduce any effects of the genetic background, in this experiment we crossed a susceptible line 22a to one of the resistant recombinants from the previous experiment. Out of 2112 individuals, 133 were recombinants in a 6cM interval containing this region. Initially 28 of these were used to create lines homozygous for the recombinant chromosomes. Again individuals from each line were injected with the virus and genotyped, which reduced the region to 298kb (Fig 1C; Wilcoxon Rank Sum Test: *W* = 176, *P* = 3.8x10^-5^). We then returned to the remaining recombinant lines, and used these to generate 8 homozygous recombinants in this reduced region. Again these lines (along with 7 lines from before) were genotyped and phenotyped. This new information allowed the identification of an 89kb region containing the candidate gene (Fig 1D; Wilcoxon Rank Sum Test: *W* = 56, *P* = 0.0013).

**Fig 2:**
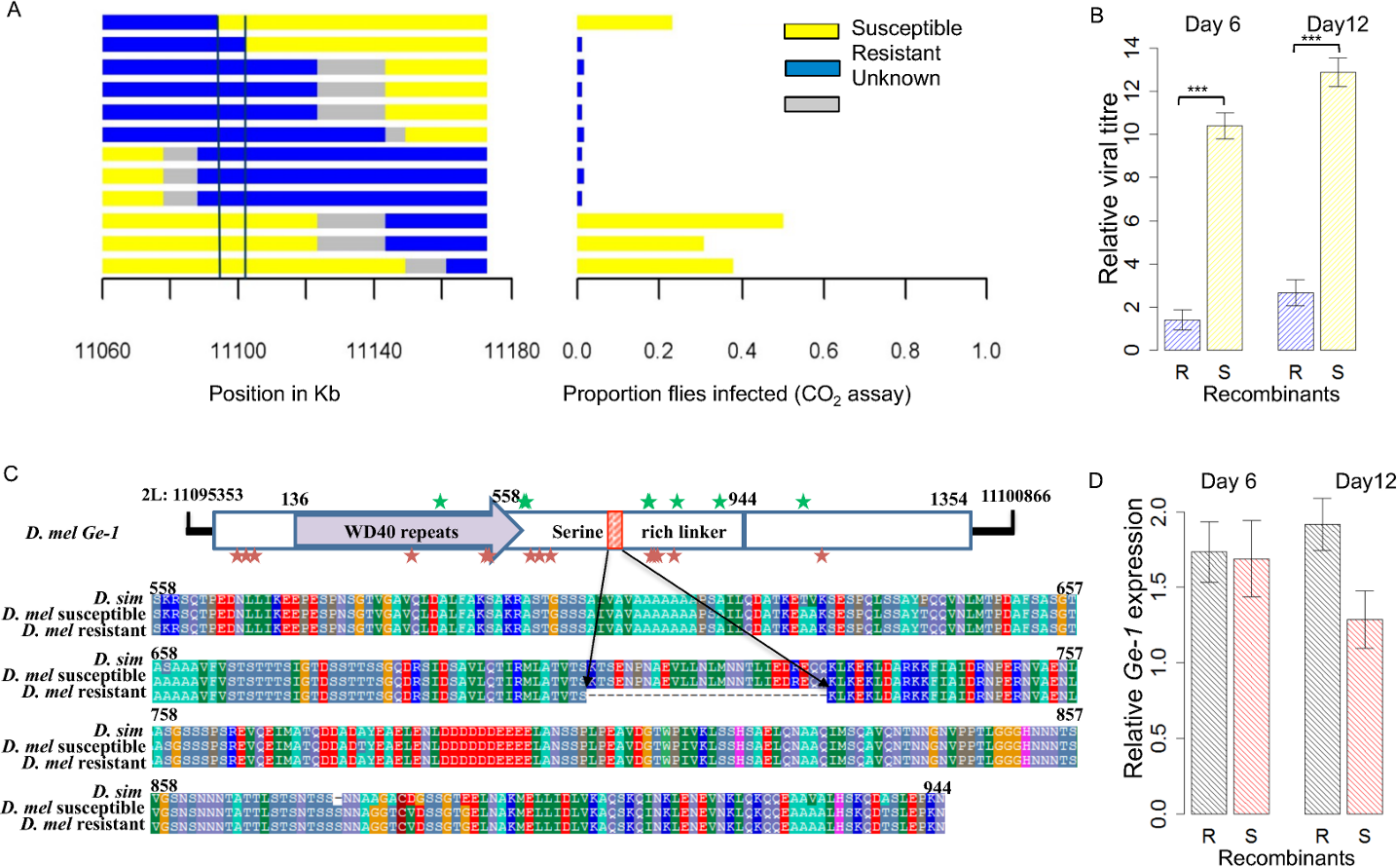
Resistance assay of resistant and susceptible recombinants. (A) Recombinants (left) were selected in the region mapped in Fig 1 using two eye-colour markers, injected with sigma virus and tested for paralysis after exposure to CO_2_. This identified an ~8 kb region containing a whole gene called *Ge-1* and the flanking 3’ untranslated regions of the genes *CG4705* and *Reps*. (B) relative viral titre in 20 resistant and 16 susceptible recombinants at 6 days post infection and 12 days post infection. (C) Domain organization of *Ge-1* (adapted from [38]). The C-terminal domain is highlighted in blue with the highly conserved C-terminal region in dark blue. Red indicates a 26 amino acid deletion that differs between the resistant and susceptible alleles. Stars represent non-synonymous differences between *D*. *melanogaster* and *D*. *simulans*. Red stars represent changes that occurred on the lineage leading to *D*. *melanogaster* and green stars represent changes on the lineage leading to *D*. *simulans*. Serine-rich linker sequences from *D*. *simulans* and *D*. *melanogaster* susceptible line 22a and resistant line EME are shown. (D) *Ge-1* expression in 20 resistant and 16 susceptible recombinants (described in Fig 2B) at 6 days post infection and 12 days post infection. Error bars are standard errors. Viral RNA loads and gene expression were measured by quantitative PCR and standardised to the reference gene *Actin5c*.

Generating recombinants in an 89kb region using this approach was not feasible, thus we turned to transposable elements carrying visible markers to select new recombinants. We chose two transposable element lines, each homozygous for an EP element that flanked our region of interest. These elements were combined on a single chromosome to generate a ‘2EP’ line [36] which was susceptible to the DMelSV (100% infected). As these elements carry visible eye-colour markers, recombinants between our resistant chromosome and the 2EP line can be detected from their light orange eye colour (the non-recombinant resistant and susceptible flies have white or dark orange eyes respectively). This approach was used to generate 12 recombinant lines. Again the lines were assayed for resistance to DMelSV and genotyped for several markers across the 89kb region, which reduced the region to just under 8kb (2L: 11094733–11102848, BDGP5; Fig 2A, Wilcoxon Rank Sum Test: *W* = 32, *P* = 0.0063). This region contains a whole gene called *Ge-1* and the flanking non-coding regions upstream of the genes CG4705 and *Reps*. Ge-1 has been shown involved in RNA degradation and the formation of processing bodies (P bodies) in animals and plants [37–40]. It acts as a bridge between Decapping protein 1 and Decapping protein 2, which remove the 5’ cap from mRNA (“decapping”). This results in the RNA molecule being rapidly degraded by exonucleases [39].

In all of the experiments above we have used the symptom of paralysis after CO_2_ exposure to map resistance. To check that *Ge-1* is affecting the viral load rather than CO_2_ sensitivity itself, we used quantitative PCR to measure viral titres in 20 of the resistant and 16 of the susceptible recombinant lines after they had been injected with the DMelSV. We found that six days after the injection, there was approximately a 471–fold lower viral load in resistant lines compared to susceptible lines (Fig 2B; *F*_*1*,*76*_ = 137.5, *p*<2.2e^-16^), and after 12 days this rose to a 1168–fold difference (Fig 2B; *F*_*1*,*97*_ = 125.8, *p*<2.2e^-16^).

### The resistant allele of *Ge-1* contains a 26 amino acid deletion

To identify the mutations that could be responsible for resistance, we sequenced this entire region (2L: 11094733–11102848) in the original resistant and susceptible line. The two sequences differed by 47 SNPs and 1 indel. The indel is 78bp long in the 5^th^ exon of *Ge-1* (2L: 11097925–11098002), and reduces the length of the serine-rich linker region of the protein by 26 amino acids, with the resistant allele being the shorter of the two (Fig 2C). The homolog of this gene in *D*. *simulans* encodes the full length protein, suggesting that a deletion mutation has occurred in this region on the resistant chromosome. The SNPs are spread across the 3’ UTR of *Reps* and *CG4705* and all of *Ge-1*, with only two found in intergenic regions (S1 Table). To examine whether the difference seen in viral resistance could be due to a change in the expression of *Ge-1*, we used quantitative PCR to measure its expression in 20 resistant and 16 susceptible recombinant lines that were used in the viral load measurement. We found that there was no significant difference in gene expression in the resistant and susceptible lines both six days and twelve days after they had been injected with the virus (Fig 2D; day6: *F*_*1*,*76*_ = 0.0189, *p* = 0.891; day12: *F*_*1*,*97*_ = 6.109, *p* = 0.015).

We estimated the frequency of the 26 amino acid deletion in natural populations from the USA and Africa. We scored the presence or absence of the deletion by PCR in the 189 inbred lines from North Carolina that comprise the DGRP panel [41]. We found that only two of the lines contained the deletion. We also looked for the deleted allele in genome sequences from several African populations (319 alleles of *Ge-1*) [42], and none of the lines contained the deletion.

In a separate experiment, we have injected all of the DGRP lines with the DMelSV and measured the proportion of flies that were infected 13 days later using the CO_2_ assay [17]. We found that the two lines with the deletion lines were both very resistant to the DMelSV (4% of flies in line 153 and 8% in line 361 were infected after injection, compared to an average of 40.6%), but this difference was not significant (S1 Fig; MCMCglmm: *p* = 0.092). We also tested all of the other polymorphisms in the 8kb region for an association with resistance and found that only one SNP, located in the 3’ UTR of *Reps*, was significant (MCMCglmm: *p*<0.001).

### Knocking down *Ge-1* expression increases susceptibility to DMelSV

**Fig 3:**
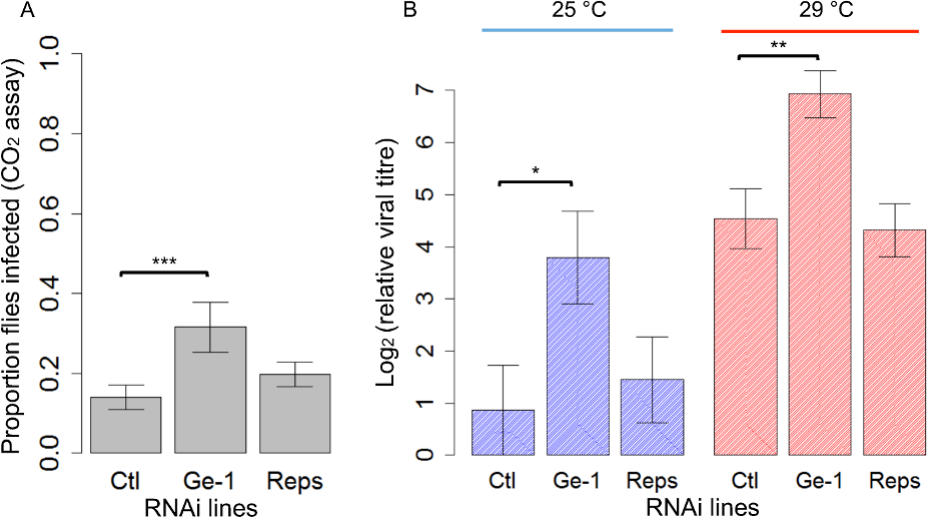
The effect of knocking down *Ge-1* and *Reps* by RNAi on susceptibility to DMelSV. (A) Proportion of flies that were paralysed after exposure to CO_2_ in control, *Ge-1-RNAi* and *Reps-RNAi* lines. Flies were kept at 25°C. (B) Viral titres in control, *Ge-1-RNAi* and *Reps-RNAi* lines standardised to reference gene *Ef1alpha100E*. This experiment was repeated at 25°C and 29°C. Error bars are standard errors.

In order to test if *Ge-1* or *Reps* is involved in sigma virus resistance in *D*. *melanogaster*, we used RNAi to knock down the expression of the two genes. These flies did not have the *Ge-1* deletion. As we found that knock-downs in flies reared at 25°C are lethal, the flies were reared at a low temperature (18°C) where *Gal4* drivers are normally inefficient before being transferred to a higher temperature (25°C or 29°C). *Ge-1*-RNAi flies showed a higher proportion of flies infected after exposure to CO_2_ than the control (Fig 3A; Generalized Linear Model: *|z|* = 3.391, *P*<0.001) while *Reps*-RNAi flies had a similar proportion of flies infected to the control (Generalized Linear Model: |*z*| = 0.951, *P* = 0.34). We also measured the viral titres to see whether the resistance is due to reduction in viral replication in flies. We found that the viral load in the *Ge-1*-RNAi line was ~8-fold higher than in the control (|*t*| = 2.336, *P* = 0.02) while *Reps*-RNAi flies had a similar viral load to the control (Fig 3B blues bars; |*t*| = 0.490, *P* = 0.627). We repeated the RNAi test and kept injected flies at 29°C. This time the viral titre in *Ge-1*-RNAi line was ~5-fold higher than in the control (|*t*| = 3.281, *P* = 0.002) while the *Reps*-RNAi flies had similar viral loads to the control (Fig 3B red bars; |*t*| = 0.282, *P* = 0.779). The knockdowns in all these flies were likely inefficient, as qPCR on *Reps* and *Ge-1* RNA levels was not significantly different from the controls.

### Deleting the 26 amino acid region of *Ge-1* in transgenic flies makes them resistant to DMelSV

**Fig 4:**
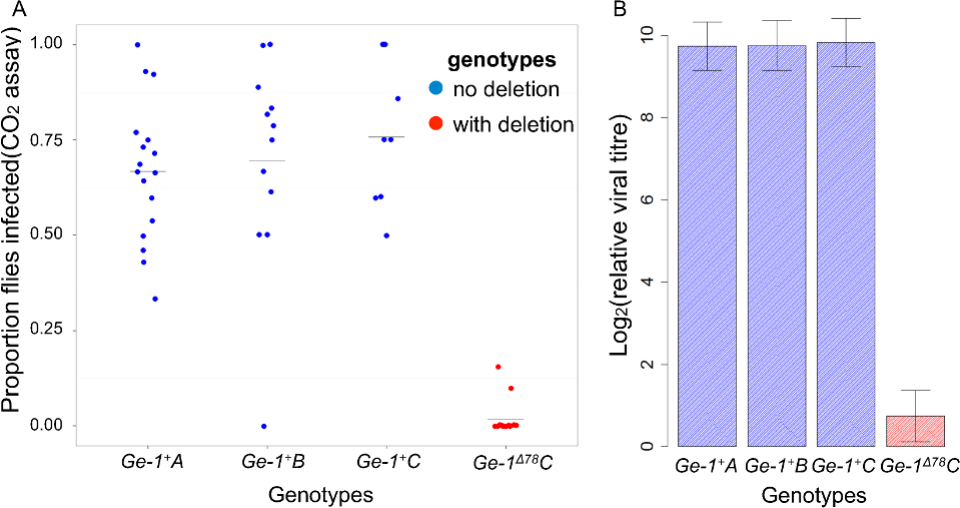
Transgenic flies with the 26 amino acid deletion in *Ge-1* are resistant to DMelSV. Flies were transformed with BAC clones carrying *Ge-1* that differs only in the presence (red) or absence (blue) of the deletion. The four genotypes are independent transformants. (A) The proportion of flies that was paralysed or dead after exposure to CO_2_. Each point is a vial of flies. Horizontal bars represent the mean of each line. (B) Mean viral titre in transgenic lines. Viral RNA loads were measured by quantitative PCR and standardised to the reference gene *RpL32*. Error bars are standard errors.

To test whether the 26 amino acid deletion in *Ge-1* is causing flies to be resistant to DMelSV, we generated transgenic flies that only differ by this deletion. To do this we first took a BAC clone of the region that lacked the deletion, and used recombineering [43] to seamlessly delete the 26 amino acids in *E*. *coli*. We then inserted two forms of the BAC into identical positions on the 3^rd^ chromosome using the *phiC31* integrase system [44], to generate flies that express the two alleles of the gene under the control of the same natural promoter. In total we constructed four independently transformed transgenic lines, one with the deletion (*CH322-Ge-1* ^*Δ78*^ *C*) and three without (*CH322-Ge-1* ^*+*^ *A*, *CH322-Ge-1* ^*+*^ *B* and *CH322-Ge-1* ^*+*^ *C*). These transgenic lines were then crossed to a *Ge-1* null mutant, *dGe-1* ^*Δ5*^ */Cyo*, so that only the transgene is expressed. The *dGe-1* ^*Δ5*^ mutation is homozygous lethal [37], and the insertion of either allele of *Ge-1* on the 3^rd^ chromosome complemented this lethal effect, allowing *dGe-1* ^*Δ5*^ homozygotes to be generated (*dGe-1* ^*Δ5*^ *;CH322-Ge-1* ^*Δ78*^ *C*, *dGe-1* ^*Δ5*^ *;CH322-Ge-1* ^*+*^ *A*, *dGe-1* ^*Δ5*^ *;CH322-Ge-1* ^*+*^ *B* and *dGe-1* ^*Δ5*^ *;CH322-Ge-1* ^*+*^ *C*). Transgenic flies carrying the deletion in *Ge-1* are highly resistant to DMelSV. Following injection with DMelSV, only 1.6% of flies from the line with the deletion showed the symptoms of paralysis and death after exposure to CO_2_ compared to 69.6% of the flies in the three lines without the deletion (Fig 4A; Generalized Linear Mixed Model: |*z|* = 7.350, *P*<<0.001). Similarly, viral titres were 512-fold higher in the transgenic line with the deletion than the three lines without the deletion (Fig 4B; *F*_*1*,*56*_ = 156.9, *P*<<0.001).

In order to test whether *Ge-1* expression differs in transgenic lines, we measured *Ge-1* expression in *dGe-1* ^*Δ5*^ *;CH322-Ge-1* ^*Δ78*^, *dGe-1* ^*Δ5*^ *;CH322-Ge-1* ^*+*^ (mix of A, B and C) flies. We didn’t detect any significant difference in *Ge-1* expression in these two genotypes (S1 Fig; *F*_*1*,*37*_ = 0.008, *P* = 0.92). In addition, we measured *Ge-1* expression in DMelSV injected flies and controls that were injected with Ringer’s solution. In both transgenic lines, we found no significant difference between virus injected and Ringer’s injected flies (S1 Fig; *Ge-1* ^*+*^: *F*_*1*,*18*_ = 2.32, *P* = 0.15; *Ge-1* ^*Δ78*^ *C*:*F*_*1*,*17*_ = 0.403, *P* = 0.53). These results indicate that *Ge-1* expression does not differ in the resistant and susceptible transgenic flies and DMelSV infection does not induce *Ge-1* expression.

### *Ge-1* resistance to DMelSV is not dependent on the siRNA pathway

**Fig 5:**
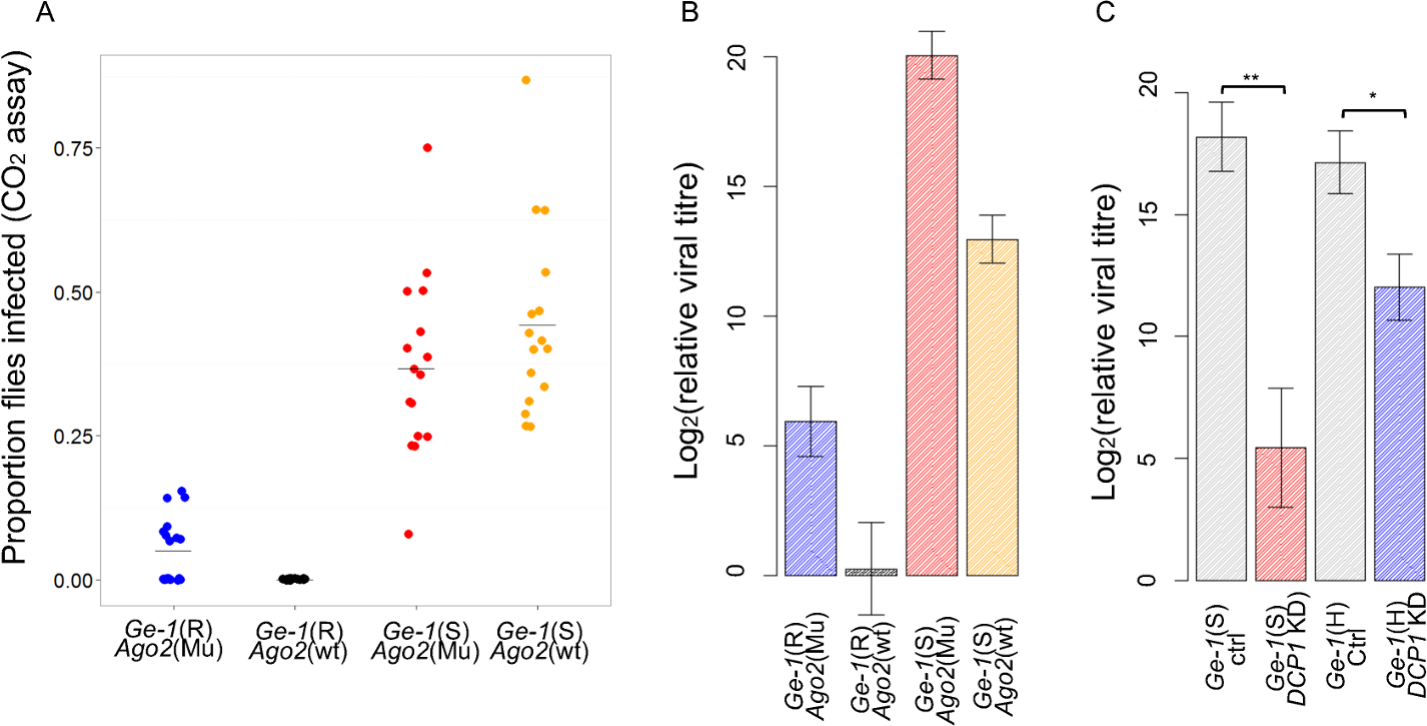
The effect of *Ago2* and *DCP1* on susceptibility to DMelSV. (A) and (B) are the effect of *Ge-1* on susceptibility in *Ago2* ^*51B*^ mutant *flies*. Blue represents flies with the resistant *Ge-1* allele and *Ago2* mutant, black the resistant *Ge-1* allele and *Ago2* wild-type, red the *Ge-1* susceptible allele and *Ago2* mutant, and orange the susceptible *Ge-1* allele and *Ago2* wild-type. (A) CO_2_ sensitivity assay. (B) Relative viral titre estimated by quantitative PCR relative to *RpL32* was used as reference gene. (C) The effect of knocking down *DCP1* on viral titre in *Ge-1* susceptible backgrounds (red bar) and *Ge-1* heterozygous backgrounds (blue bar). Grey bars are controls for the RNAi. Error bars are standard errors.

The RNAi effector protein Argonaute 2, which is a key antiviral defence in *Drosophila*, localises to some P bodies and this localisation depends on Ge-1 [37,38,40]. To test whether *Ge-1* resistance to sigma virus is mediated by RNAi, we crossed the mutant allele *Ago2* ^*51B*^ into lines carrying the susceptible and resistant alleles of *Ge-1*. This allele of *Ago2* deletes the first two exons and is known to abolish its slicer activity [45], but it does still produce the shortest *Ago2* transcript and this may have some function [46]. In the CO_2_ sensitivity assay, the two alleles of *Ge-1* significantly affected susceptibility regardless of whether there was a functional allele of *Ago2* (Fig 5A; Wilcoxon Rank Sum Test with *Ago2* mutant: |*Z|* = 4.9, *P* = 8.99e^-7^; Wilcoxon Rank Sum Test with *Ago2* wild-type: |*Z*| = 5, *P* = 4.7e^-7^). The same pattern was found when we measured viral titre in these lines–*Ge-1* had a large effect on viral titre (Fig 5B; *Ge-1*: *F*_*1*,*37*_ = 100.82, *P*<0.0001), but this was not affected by *Ago2* (interaction Ge-1*Ago2: *F*_*1*,*37*_ = 0.3, *P* = 0.59). The *Ago2* ^*51B*^ mutants did have higher titres of DMelSV (Fig 5B; *Ago2*: *F*_*1*,*37*_ = 35.69, *P*<0.0001). Therefore, siRNA pathway does defend flies against DMelSV but *Ge-1* does not rely on the siRNA pathway to provide resistance.

### Decapping protein 1 protects flies against DMelSV

*Ge-1* is essential for forming P bodies, so we investigated the role of eight other P-body components in DMelSV resistance. As mutants tend to be lethal, we used RNAi to knock down the expression of these genes in adult flies. We found that knocking down the expression of *Decapping protein 1* (*DCP1*) resulted in increased viral load (Fig 5C; *Ge-1* susceptible background: Wilcoxon Rank Sum Test: *W* = 27, *P* = 0.009; *Ge-1* heterozygous background: Wilcoxon Rank Sum Test: *W* = 87, *P* = 0.02). The effect of knocking down *DCP1* was greater in *Ge-1* susceptible flies than in flies that were heterozygous for the two *Ge-1* alleles (Fig 5C; interaction between *Ge-1* and *DCP1*: |*t*| = 2.15, *P* = 0.03). However, we would interpret this interaction cautiously, as the *Ge-1* susceptible and heterozygous flies did not differ in their viral load, perhaps due to genetic background effects or dominance (Fig 5C). Knocking down the other genes did not have a significant effect on DMelSV titres (S2 Fig; *Edc3*, *DCP2*, *DCP1*, *GW182*, *pcm*, *me31B*, *Part-1* and *stau*; note the efficiency of these knockdowns was not checked).

### *Ge-1* does not affect susceptibility to DAV or DCV in *D*. *melanogaster*

**Fig 6:**
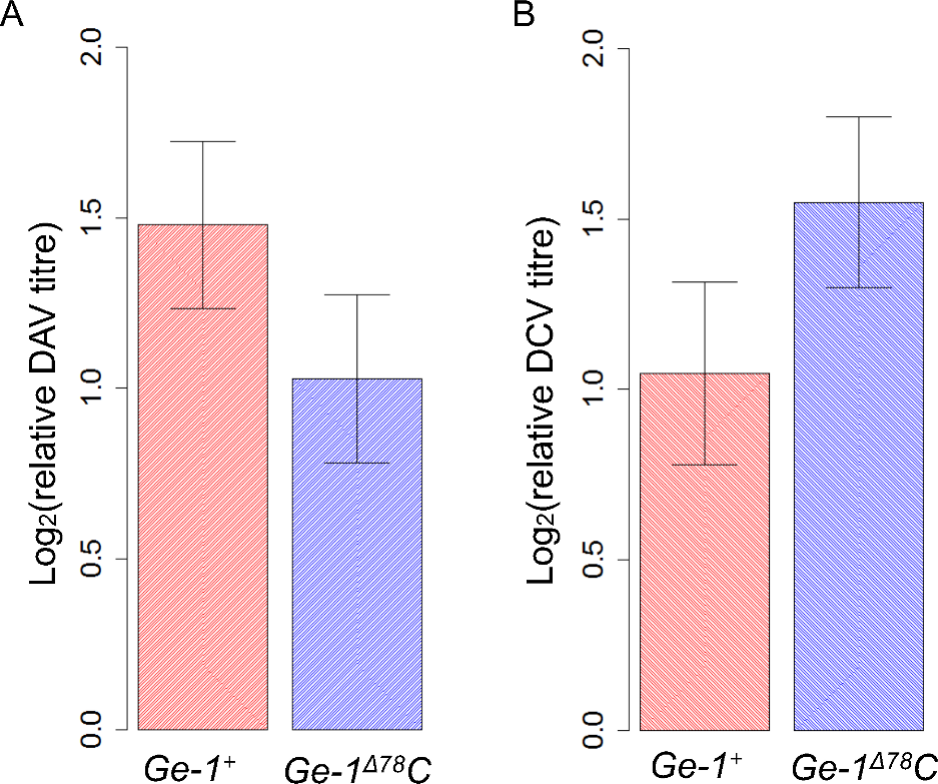
Titres of DAV and DCV in infected *Ge-1* transgenic flies. (A) Relative viral titres in DAV infected flies. (B) Relative viral titres in DCV infected flies. Error bars are standard errors.

We tested whether *Ge-1* resistance was specific to DMelSV by infecting *Ge-1* transgenic flies with *Drosophila* A virus (DAV) and *Drosophila* C virus (DCV). Both DAV and DCV are natural pathogens of *D*. *melanogaster*, and DAV infects flies in nature and laboratories widely [30,47]. Transgenic flies carrying resistant (*Ge-1* ^Δ78^C) or susceptible allele (*Ge-1* ^+^) of *Ge-1* have similar viral titres after DAV infection (Fig 6A; *F*_*1*,*16*_ = 1.685, *P* = 0.21) and after DCV infection (Fig 6B; *F*_*1*,*13*_ = 1.871, *P* = 0.19). This indicates that this polymorphism in *Ge-1* does not have an effect on DAV or DCV infections and its antiviral function is likely to be specific to sigma virus.

### The serine-rich linker of Ge-1 has evolved under positive selection in *D*. *melanogaster* and *D*. *simulans*

As changes in *Ge-1* can make flies resistant to viral infection, it is possible that it has been the target of sustained selection by viruses during evolution. To investigate this we used a McDonald-Kreitman Test to detect beneficial amino acid changes that have been fixed by natural selection during the evolution of *Ge-1* [48]. In both *D*. *melanogaster* and *D*. *simulans* we estimate that over 80% of the amino acid substitutions were fixed by natural selection (α in Table 1) and therefore had a beneficial phenotypic effect. Virus resistance is caused by a change to the serine-rich linker, and 13 of the 22 non-synonymous changes between *D*. *melanogaster* and *D*. *simulans* are located in this region (stars in Fig 2B). When we repeated the test on just the serine-rich linker we found evidence of selection on this domain in both the *D*. *melanogaster* and *D*. *simulans* lineages and no evidence of selection in the remainder of the protein (Table 1).

**Table 1:** McDonald Kreitman test on *Ge-1*.

Species	Protein region	Class sites	Polymorphism	Divergence	α	χ^2^	*p*-value
***D*. *melanogaster***	Whole protein	Synonymous	45	38			
		Non-synonymous	3	14			
		N/S	0.07	0.37	0.82	7.56	0.005
	S-rich linker (558–944)	Synonymous	19	12			
		Non-synonymous	1	7			
		N/S	0.05	0.58	0.91	6.06	0.01
	Other regions (1–557, 945–1354)	Synonymous	26	26			
		Non-synonymous	2	7			
		N/S	0.08	0.27	0.71	2.38	0.12
***D*. *simulans***	Whole protein	Synonymous	80	17			
	protein	Non-synonymous	5	8			
		N/S	0.06	0.47	0.87	12.64	<0.001
	S-rich linker (558–944)	Synonymous	19	3			
		Non-synonymous	0	5			
		N/S	0	1.67	1	14.57	<0.001
	Other regions (1–557, 945–1354)	Synonymous	61	14			
		Non-synonymous	5	3			
		N/S	0.08	0.21	0.62	1.57	0.21

Divergence was polarised to the lineage leading to *D*. *melanogaster* and *D*. *simulans*. As well as whole-protein analyses, the serine-rich region only and other domains were analysed separately. For the polymorphism data we used *Ge-1* sequences from 197 Zambian *D*. *melanogaster* genomes (only site with minor allele frequencies over 10%) [42] and 6 *D*. *simulans* genomes [66]. So that we can look separately at evolution occurring on the lineage leading to *D*. *melanogaster* and *D*. *simulans*, we reconstructed the most recent common ancestor of the sequences of *Ge-1* that are found in *D*. *simulans* and *D*. *melanogaster* using outgroups. α is the estimated proportion of amino acid changes that were fixed by natural selection.

## Discussion

We have identified a naturally occurring polymorphism in a gene called *Ge-1* that makes *D*. *melanogaster* highly resistant to the naturally occurring rhabdovirus DMelSV. When we knocked-down the expression of the susceptible allele of *Ge-1* by RNAi the flies became even more susceptible to infection, indicating that *Ge-1* is an existing restriction factor whose antiviral effects have been increased by the deletion. By modifying the sequence of the gene in transgenic flies, we identified a 26 amino acid deletion in the serine-rich linker region of *Ge-1* that is causing the resistance. This polymorphism has no effect on resistance to DAV or DCV, indicating resistance is likely to be specific to sigma or related viruses. Ge-1 acts as a bridge bringing together DCP1 and Decapping Protein 2 (DCP2), which can then remove the 5’ cap from mRNA leading to its degradation [38]. We found that DCP1 also restricts sigma virus infection in flies, suggesting that this pathway may underlie resistance. The serine-rich linker of Ge-1 has experienced strong selection during its evolution, suggesting that it may be involved in an ongoing arms race with viruses.

Ge-1 plays a central role in RNA degradation and the formation of processing bodies (P bodies) in animals and plants [37–40]. It is required for the removal of the 5’ cap from mRNA, which results in the subsequent degradation of the RNA molecule by an exonuclease in the 5’ to 3’ direction [49]. It is thought to act as a bridge between two of the key molecules involved the decapping process, with its C-terminal domain interacting with the protein DCP2 and its N-terminal domain interacting with DCP1 [39]. The C-terminal domain of Ge-1 also results in the localization of the protein to P bodies, which are sites in the cytoplasm where many of the enzymes involved in RNA degradation are localized. Ge-1 not only localises to P bodies, but it is also required for P-body stability and formation, probably due to its role as a scaffold protein [38,39].

P bodies can play both pro- and anti-viral roles [50]. In some cases viruses disrupt P body formation, presumably to prevent viral RNA being degraded. For example, P body components act as restriction factors for influenza A virus, and the viral NS1 protein in turn disrupts the formation of P bodies [51]. Similar interactions also occur between insects and their viruses. In *Drosophila* the dicistrovirus CrPV disrupts P bodies [52]. Furthermore, several P body components involved in mRNA decapping have an antiviral effect against the bunyavirus Rift Valley fever virus (RVFV) [53]. This is thought to be because bunyaviruses “cap-snatch” their 5’ cap from host mRNAs, and the decapping process reduces the availability of 5’ caps for viral replication [53]. As Rhabdoviruses do not cap-snatch, this mechanism cannot explain our results. Other viruses have co-opted P bodies for their own benefit. This is the case for West Nile virus, which recruits various P body components to its replication centres, and knocking down the expression of these proteins results in lower viral titres [54].

There are two possible ways in which Ge-1 could confer resistance. First, it could be used in some unknown way by DMelSV during the viral replication cycle, and the resistant allele of the gene may interfere with this process. Second, it could play an existing antiviral function that is made more efficient. We found that knocking down the expression of the susceptible allele of *Ge-1* further increased the susceptibility of flies, indicating that the polymorphism that we identified is increasing the effectiveness of an existing restriction factor.

We demonstrated that a 26 amino acid deletion in *Ge-1* is the cause of increased resistance to DMelSV by modifying the gene in transgenic flies. We ensured the gene was under the control of its natural promoter by modifying BAC clones of this region of the *Drosophila* genome, and these BACs were then inserted into a fly line that carried a null allele of *Ge-1*. This deletion removes 26 amino acids from the middle of the protein, which is a flexible linker that lies between two structured domains. It does not alter the sequence of the domains that interact with DCP1 and DCP2, or the region required for P body formation [38]. It is therefore possible that the deletion may affect the protein’s role as a scaffold by changing the conformation of the protein complex.

We investigated two possible ways in which Ge-1 might be affecting the susceptibility of flies. The primary antiviral defence of insects is RNAi, and the RNAi-Induced Silencing Complex (RISC) localises to P bodies [55,56]. However, our results show that *Ge-1-*based resistance does not require Ago2, and therefore is independent of RNAi. Therefore, a likely hypothesis is that resistance relies on the destruction of viral genomic or messenger RNA in an RNAi-independent way in P bodies. Consistent with this we found that knocking down the expression of DCP1 increases DMelSV titres in flies. As the polymorphism affecting resistance is in the serine-rich linker that forms a bridge between DCP1 and DCP2, it seems likely that Ge-1-based resistance involves the decapping complex.

By looking at the *Ge-1* sequence in *D*. *simulans*, we found the ancestral state of *Ge-1* is the susceptible allele. This fits with an evolutionary arms race in which hosts are continually evolving new defences against pathogens. Consistent with this, we found that the protein sequence of Ge-1 has been subject to long term positive selection. While our analysis demonstrates that many of the amino acid substitutions that have occurred in the *D*. *melanogaster* lineage provided a selective advantage to flies, further work would be needed to demonstrate this was linked to the antiviral function of Ge-1 (especially given the mutation that gave rise to resistance was a deletion not a substitution). Nonetheless, studies of other genes involved in antiviral immunity, such as those in the siRNA pathway, have found that they are also evolving rapidly under positive selection [57,58]. Therefore, selection by viruses may frequently be an important force in the molecular evolution of genes involved in antiviral defence.

The resistant allele of *Ge-1* is currently rare, being found in just 1% of the North American flies we tested and none of the African flies. There are multiple possibilities that may contribute to it being a rare polymorphism. First, it could be a relatively new mutation which has not had time to spread among *D*. *melanogaster* populations. Second, we examined the frequency of the polymorphism in North America and Africa but the resistant allele was isolated from France and it may be more common there. Finally, it could be costly. For example, if it disrupts mRNA degradation it might have many pleiotropic effects on other traits. This could maintain the allele at a low frequency if there is only a net benefit to being resistant at certain times or places where the viral prevalence is high. Alternatively, costs may maintain the allele at low frequency due to heterozygote advantage or negatively frequency dependant selection.

Our results show that studying naturally occurring polymorphisms that increase resistance to infection provides a valuable alternative to studying immunity through artificial mutations that reduce resistance. Not only can this approach identify novel forms of antiviral defence, but it also allows us to understand how resistance to infection evolves. This is important as evolved defences may not always involve the classical immune response.

## Materials and Methods

### Recombinant mapping fly lines and crosses

Susceptible (22a) and resistant (EME) fly lines were provided by Didier Contamine. The 2^nd^ chromosome in the EME stock carries the resistant allele of the gene *ref(2)M* [34], and the other two chromosomes are from a susceptible ebony stock. To map the resistance genes we crossed the resistant and susceptible parental stocks and created lines that carried homozygous recombinant chromosomes. The female F1 progeny of a cross between EME and 22a were crossed to a balancer stock *SM5*,*Cy/Pm*. In the next generation we selected individual *SM5*,*Cy/+* males and crossed these back to the balancer. A few days after setting up this cross we removed the male parents from the vial and genotyped them using molecular markers flanking the region that we knew contained the resistance gene (S2 Table). This allowed us to discard all the lines that had not recombined in this region and only retain the informative recombinants. In the next generation we crossed sibling *SM5*,*Cy/+* flies, and then selected for homozygous recombinants in the subsequent generation. Using this approach we initially generated 33 lines that had recombined between insertion/deletion (indel) markers at 36cM and 52cM on the 2^nd^ chromosome. We then repeated the experiment to produce recombinants between markers at 42cM and 47cM.

To select recombinants in even smaller regions we used phenotypic markers flanking the region of interest rather than molecular markers. First, mapping lines were generated by crossing two *P*-element lines that flanked the region of interest to create a chromosome carrying both *P*-elements. These elements both carried the mini-white gene, and flies that carry a single heterozygous element have lighter coloured eyes than flies carrying two heterozygous elements [36].The lines w*;P{EP}2377 and w*;P{EP}2478 were combined to the same chromosome to generate the 2^nd^ chromosome mapping line (2EP-2). This was then crossed to one of the resistant recombinant lines (M34) that had been generated in the experiment described above. 12 homozygous 2^nd^ chromosome recombinant lines were generated using the balancer SM5,Cy/+ and the crossing scheme described above.

### Genotyping

DNA was extracted using either Chelex resin (Sigma-Aldrich) [59] or a Tissue Genomic DNA kit (Metabion, Munich) using manufacturer protocols. Genotyping of each location was done using microsatellites, Indels, SNP-specific primers or via sequencing (S2 Table). The PCR reaction consisted of incubation at 95°C (5min), followed by 30 cycles of 95°C (30 sec), 55°C (20 sec) and 68°C (1 min per kb), then 68°C (8min). Short PCR products were run on 2% agarose gels, while larger products were run on 1% agarose gels to score length differences (indels and microsattelites). PCR products for sequencing were cleaned up by incubating with the restriction enzyme ExoI and Shrimp Alkaline Phosphotase (SAP) at 37°C for 1hr, followed by 15 min incubation at 72°C to deactivate the enzymes. The sequencing reaction consisted of 25 cycles of 95°C (30 sec), 50°C (20 sec) and 60°C (4 min) using BigDye reagents (ABI). Sequencing was carried out at either Source Bioscience Life Sciences (Cambridge) or The Genepool (Edinburgh).

### Sequencing and genotyping candidate regions

DNA for sequencing was extracted using either Tissue Genomic DNA kit (Metabion, Munich) or DNeasy 96 Blood & Tissue Kit (Qiagen). The 8 kb region identified on chromosome 2 (2L: 11094733–11102848, BDGP5) was sequenced for lines EME and 22a. Primer pairs were designed to amplify these regions in overlapping fragments (S2 Table), and the sequencing was performed as described above. Diagnostic PCR primers were designed to genotype flies for a deletion in *Ge-1*. The forward primer *Ge-1* Indel 1F (5’ AGCGTCAAGCTTTTCCTTCA 3’) and the reverse primer *Ge-1* Indel 1R (5’ CACCAGCGGTCAGGATAGAT 3’) were used to establish presence or absence of the 78bp deletion in *Ge-1*.

### *D*. *melanogaster* sigma virus (DMelSV)

The Hap23 strain of DMelSV was extracted from an infected line of *D*. *melanogaster* (Om) using the protocol described in Magwire *et al*. 2011[17].

### Fly infection and measuring resistance

Female *D*. *melanogaster* were injected in the abdomen with sigma virus either by blowing virus through a glass needle connected with a hose until slight extension of the proboscis was observed (only for recombinant mapping experiment) or by using Nanoject (Drummond Scientific) to inject 69nl virus suspension (all other experiments). Injected flies were tipped onto new media every two days before they were tested for infection. Flies infected with the DMelSV become paralysed and die on exposure to CO_2_. To test for this symptom of infection, the flies were exposed to CO_2_ for 15 minutes at 12°C 10–16 days post infection. The exact gassing date for each experiment was carefully picked by pilot CO_2_ exposure. Flies were given 2 hours to recover from the CO_2_ and then the number of dead or paralyzed individuals was counted as well as the total number of individuals in each vial.

In the recombinant mapping experiment, four replicate vials each containing approximately 20 flies were used in each experiment except for the first round of recombinant assay (one replicate) and were used in the CO_2_ sensitivity assay. In the 25°C RNAi experiment, 29–40 replicate vials for each cross were injected with virus. 14–25 were used in the CO_2_ sensitivity assay and the remaining 15 vials were used to measure viral titre (see details below). In the 29°C RNAi experiment, 24 copies of each cross were injected with sigma virus and all of them were used to measure viral titre. In the transgenic fly experiment and the *Ago2* dependency experiment, 30–32 replicate vials each containing approximately 15 flies were injected with virus. 15–18 vials per line were assayed for resistance to sigma virus by measuring CO_2_ sensitivity and the remaining 15 vials were used to measure viral titre.

### Quantitative RT-PCR

RNA was extracted from 8–15 individuals from each line at day 6 and/or day 12 post sigma virus injection using TRIzol (Invitrogen Corp, San Diego) following the manufacturer’s instructions. In recombinant mapping experiment, viral RNA load was measure using SensiFAST SYBR & Fluorescein Kit on cDNA template. RNA was reverse transcribed into cDNA using MMLV (Invitrogen) or GoScrip Reverse Transcriptase (Promega) and random hexamers (Sigma). Viral load was determined using quantitative PCR using fluorescein and the primers DmelSV_F1 (5’ TTCAATTTTGTACGCGGAATC 3’) and DmelSV_R1 (5’ TGATCAAACCGCTAGCTTCA 3’), which amplify a region of the viral genome spanning one gene and the 5’ linker (and therefore amplify genomic RNA but not mRNA) in mapping experiments [17]. *Ge-1* expression was measured using the forward primer qGe-1_F2 (5’ TCTTTGTGTCTCGAGCATGG 3’) and the reverse primer qGe-1R3 (5’ GAGCAAGCAATTTCTGGATACTT 3’). Expression of *Actin 5C* was used as a reference using the primers qActin5c_for2 (5’ GAGCGCGGTTACTCTTTCAC 3’) and qActin5c_rev2 (5’ aagcctccattcccaagaac 3’) [17]. In RNAi and transgenic experiments, viral load was measured using a QuantiTect Virus+ROX Vial Kit (QIAGEN) on RNA template. Dual-labelled probe [FAM] TGTGCCAAGTCTGTAATCCTGCTA [NFQ-MGB] and primers DMelSV_F (5’ CCGACTACAAATGCTATATG 3’), DMelSV_R (5’ CAGGTATTAGAGGCTTCTTA 3’) were used to amplify DMelSV genomic RNA. The amount of viral RNA and gene expression were standardised to twohousekeeping genes, *Ef1alpha100E* or *RPL32*, using the ΔΔCt (critical threshold) method (see below). *Ef1alpha100E* was amplified using probe [FAM] ATCGGAACCGTACCAGTAGG [BHQ3] and primers Ef1a100E_FW (5’ GGACGTCTACAAGATC 3’) and Ef1a100E_RV (5’ TCTCCACAGACTTTAC 3’). *RPL32* was amplified using probe [VIC] ACAACAGAGTGCGTCGCCGCTTCAAGG [NFQ-MGB] and primers RPL32_FW (5’ TGCTAAGCTGTCGCACAAATGG 3’) and RPL32_RV (5’ TGCGCTTGTTCGATCCGTAAC 3’). We performed two or three technical replicates of each PCR and used the mean of these in subsequent analyses. We calculated the PCR efficiency by using a dilution series. Using this approach we found that the actin PCR is 93% efficient, the virus PCR using fluorescein is 96% efficient, the *Ge-1* PCR is 102% efficient, the virus PCR using probe is 98% and the *RPL32* PCR is 96%.

### RNAi

Fly strains UAS-*Ge-1*-RNAi (*y*, *w* ^*1118*^;P{KK102275}VIE-260B, KK 106687), UAS-*Reps*-RNAi (*y*, *w* ^*1118*^;P{KK101677}VIE-260B, KK 110704) as well as a control strain *y*, *w* ^*1118*^ *;P{attP*,*y* ^*+*^,*w* ^*3-*^ *}* (60100) were bought from VDRC Stock Center [60]. A ubiquitously expressed *Gal4* driver under the control of the *daughterless* promoter w*;P{GAL4-da.*G32}UH1* was crossed to the UAS strains and the control strain to induce the knock down effect. Four replicates were set up for each cross and flies were reared in cornmeal bottles with live yeast at 18°C where *Gal4* drivers are inefficient (efficient knock-downs are lethal). Two- to three-day old mated F1 females were injected with DMelSV for each cross and injected flies were kept at 25°C to induce an efficient knock-down effect before assaying for resistance. We later repeated this experiment and kept the injected flies at 29°C for a more efficient knock-down effect. The *Ge-1*-RNAi construct is 345 bp long and targets exon 9 of *Ge-1*. The *Reps*-RNAi construct is 360 bp long and targets exon 4 of *Reps*.

Since *Ge-1* is essential for forming P bodies, we also tested the effect of knocking down other P-body genes by RNAi on DMelSV susceptibility [60]. We knocked down 8 P-body genes: *Edc3* (*CG6311*, *w* ^*1118*^ *;P{GD11886}v30149*, GD30149), *DCP2* (*CG6169*, P{KK101790}VIE-260B, KK105130), *DCP1* (*CG11183*, *P{KK101204}VIE-260B*, KK105638), *GW182* (*CG31992*, *P{KK101472}VIE-260B*, KK103581), *pcm* (*CG3291*, *P{KK108511}VIE-260B*, KK105739), *me31B* (*CG4916*, w1118; *P{GD11470}v49378*, GD49378), *Part-1* (*CG5208*, *P{KK104961}VIE-260B*, KK100872) and *stau* (*CG5753*, *P{KK108121}VIE-260B*, KK106645) in *Ge-1* susceptible background and *Ge-1* heterozygous background separately. *Daughterless Gal4* driver was crossed to *Ge-1* resistant and susceptible recombinants to generate two *Gal4* drivers with resistant or susceptible *Ge-1* backgrounds. UAS lines were then crossed to two *daughterless Gal4* drivers and kept at 18°C where *Gal4* drivers are inefficient (efficient knock-downs are lethal). Because UAS-RNAi lines contain susceptible *Ge-1* allele, so the crosses result in flies with susceptible *Ge-1* background or heterozygous *Ge-1* background. Two- to five-day old mated F1 females were injected with DMelSV for each cross and injected flies were kept at 25°C to induce an efficient knock-down effect before assaying for resistance. Infected flies were transferred to new food every three days and were homogenized at day12 post infection for RNA extraction. Note that the design of the crosses means that there are 4 different genetic backgrounds (the GD and KK RNAi lines crossed to the two different Gal4 driver lines). Within these four genetic backgrounds, the flies should be genetically identical except for the RNAi construct.

### Generating transgenic flies using recombineering

*Drosophila* P[acman] Bacteria Artificial Chromosomes (BACs) clone CHORI-322-120M19 covering fly genome 2L: 11090119–11111928 was obtained from BACPAC Resources Centre (BPRC) [61]. This BAC contains the susceptible allele of *Ge-1* which doesn’t have the deletion. Recombineering was carried out to replace the susceptible allele of *Ge-1* in the BAC with a resistant allele containing the deletion through homologous recombination [43]. DNA was extracted from the resistant stain EME to use as a template for amplifying the resistant allele of *Ge-1*. A fragment containing a region of *Ge-1* with the deletion was amplified using the primers Ge-1_newrc_F1 (5’ TATCTCCTGCACCTCTCGAC 3’) and Ge-1_newrc_R1 (5’ CTGCCTGCACGAG TGGAA 3’). The *GalK* targeting cassette was amplified from bacterial strain pgalK. Phusion High Fidelity polymerase (NEB) and the primers: Ge-1_galK_F (5’ *CGTCCTGTGTGGCCATTATCTCCTGCACCTCTCGACTCGGACTCGAACTGCCGCT-*CCTGTTGACAATTAATCATCCGCA 3’), Ge-1_galK_R (5’ *GGAAGCCACATGATGGCAAAAA-AGTCTGCTCTCTCTTTTTCCTCGACAATATCAACAAG*TCAGCACTGTCCTGCTCCTT 3’) were used in PCRs. The sequences in italics were homologous sequences to the *Ge-1* gene.

To transform flies with the BAC clone, the BAC carrying the resistant allele of *Ge-1* was injected into embryos of a fly strain containing the 3^rd^ chromosome *attp* site: *M(eGFP*, *vas-int*, *dmRFP)ZH-2A;;M(attP)ZH-86Fb* [62]. The original BAC was also injected into the same strain as a control. Since the BAC contains a mini-white gene, flies emerged from injected embryos were crossed to a white-eye double balancer *w* ^-^;*If/Cyo;TM6B/MRKS* and successful transformants were picked by their red-eye phenotype. Balanced transformants were crossed to their siblings and generated homozygotes with balanced 2^nd^ chromosome.

### Testing whether *Ge-1*-based resistance is *Ago2* dependent

*Ago2* mutant Ago2^51B^ was kindly provided by Dr. Maria Carla Saleh (Institute Pasteur). Two recombinants 47 (resistant) and 16 (susceptible) generated above were chosen to cross to an *Ago2* mutant strain Ago2^51B^ and generated two homozygous lines: *47; Ago2* ^*51B*^ and *16; Ago2* ^*51B*^. 30 vials containing 15 two- to five-day old females were infected with sigma virus for each line. The same number of flies were infected for strain 47 and strain 16 as controls. Infected females were kept at 25°C and maintained on cornmeal food.

### Infecting *Ge-1* transgenic flies with other viruses

We also infected the transgenic flies carrying resistant allele (*Ge-1* ^Δ78^C) and susceptible allele (*Ge-1* ^+^) of *Ge-1* with two other viruses extracted from *D*. *melanogaster*: DAV and DCV (TCID_50_ = 5x10^8^) [63]. For line *Ge-1* ^Δ78^C, 10 vials of 15 mated females were pricked with either DAV or DCV and kept in 25°C. 10 vials of infected females of *Ge-1* ^*+*^ (*Ge-1* ^*+*^ *A*, *Ge-1* ^*+*^ *B* and *Ge-1* ^*+*^ *C*) were also pricked with viruses and kept in 25°C. DAV infected flies were collected 3 days post infection and DCV infected flies were collected 2 days post infection for RNA extraction.

### Statistical analysis

Our data from the infection experiments consists of numbers of infected and uninfected flies, which we treat either as a proportion analysed with non-parametric statistics or as a binomial response in a generalized linear mixed model. The parameters of the model were estimated using the R library MCMCglmm [64], which uses Bayesian Markov chain Monte Carlo (MCMC) techniques. To test for an association between resistant genes and resistance to sigma virus in DGRP, we fit the model:

$$v_{i,j}={logit}^{-1}(X_{i}^{T}\beta +\alpha_{j}+\varepsilon_{i,j})$$

Where ν_*i*,*j*_ is the probability of flies in vial *i* from line *j* being infected. β is a vector of the fixed effects of *ref(2)P* genotype, *doc1420* of *CHKov1* genotype (two genes known to affect sigma virus resistance [17,19]), and X_*i*_^T^ is a row vector relating the fixed effects to vial *i*. α_*j*_ is a random effect of line *j*. The residual, ε_*i*,*j*,_ allows over-dispersion due to unaccounted for heterogeneity between vials in the probability of infection. We tested for the effects of a 78bp deletion in *Ge-1* and SNPs by including this as an additional fixed effect in β.

For each fly line in which we measured viral titres or gene expression by quantitative RT-PCR, we first calculated Δ*Ct* as the difference between the cycle thresholds of the gene of interest and the endogenous control. In recombinant mapping and transgenic lines’ assays, the viral titre or gene expression in resistant flies relative to susceptible flies was calculated as 2^-ΔΔCt^, where ΔΔ*Ct* = Δ*Ct*_*resistant*_ *—*Δ*Ct*_*susceptible*_, where Δ*Ct*_*resistant*_ and Δ*Ct*_*susceptible*_, are the means of the Δ*Ct* values of the resistant and susceptible lines. In RNAi, the viral titre or gene expression in RNAi lines relative to the control was calculated as 2^-ΔΔCt^, where ΔΔ*Ct* = Δ*Ct*_*RNAi*_ *—*Δ*Ct*_*control*_, where Δ*Ct*_*RNAi*_ and Δ*Ct*_*control*_, are the means of the Δ*Ct* values of RNAi lines and the control. To assess whether these differences were statistically significant, we compare Δ*Ct* in the resistant lines (RNAi lines) and the susceptible lines (control) by fitting the Δ*Ct* values in a linear model.

In P-body genes RNAi experiment, the viral titre data was not normally distributed. We first Box-Cox transformed the data using R function “boxcox” from package “MASS” [65]. Then we fitted the transformed data in a linear model to test whether ΔΔ*Ct* of RNAi lines were significantly different from controls.

To test for positive selection, we used a McDonald and Kreitman (MK) test [48] applied to 197 *D*. *melanogaster* sequences from Zambian lines from Drosophila Population Genomics Project 3 (DPGP3) [42] and 6 *D*. *simulans* sequences [66]. Substitutions were polarised along the *D*. *melanogaster* and *D*. *simulans* lineages. Consensus sequences of 197 *D*. *melanogaster* samples were downloaded from http://www.dpgp.org/. *Ge-1* sequences (2L: 11095353–11100866) were pulled out from all lines using the scripts “breaker.pl” and “dataslice.pl” written by the authors (Masking package available from http://www.dpgp.org/). Script “breker.pl” inserts line breaks every 1000 bp in all files and script “dataslice.pl” returns locus-specific FastA files when given a subset of individuals and locus information. Coding sequences of *Ge-1* from all the lines were manually aligned using BioEdit. Vertical multiple alignment (vma) files of 6 *D*. *simulans* lines were downloaded from the DPGP website. Vma files were converted into FastA files by script “VMA2FASTA” provided by the authors. *Ge-1* coding sequences were pulled out and manually aligned. *Drosophila yakuba* and *Drosophila erecta* sequences were used to infer the sequence of the most recent common ancestor of *Ge-1* in *D*. *melanogaster* and *D*. *simulans*. For any polymorphic codon, if the ancestral state is ambiguous (ie. both the *D*. *melanogaster* and *D*. *simulans* nucleotides were present in the outgroup species), we simply excluded it from the analysis. Standard MK test was carried out using McDonald and Kreitman Test (MKT) software [67]. We excluded polymorphic sites with a frequency less than 10%. Polarized 2 × 2 contingency tables were used to calculate α, which can be used as an estimate of the proportion of variants fixed under selection [68]. Statistical significance of the 2 × 2 contingency tables was determined by carrying out using a χ^2^ test.

## Supporting Information

S1 Fig Ge-1 expressions in transgenic flies. Blue bars are transgenic flies carrying susceptible Ge-1 allele and red bars are flies carrying Ge-1 resistant allele (with deletion).Error bars are standard errors.
(TIF)

S2 Fig RNAi knock-downs of P-body components. Eight genes encoding P-body components were knocked down by RNAi: *Edc3* (*CG6311*), *DCP2* (*CG6169*), *DCP1* (*CG11183*), *GW182* (*CG31992*), *pcm* (*CG3291*), *me31B* (*CG4916*), *Part-1* (*CG5208*) and *stau* (*CG5753*)). Left 10 boxes (red) are RNAi knock-downs in flies with *Ge-1* susceptible background. Right 10 boxes (blue) are RNAi knock-downs of flies that were heterozygous for the resistant and susceptible *Ge-1* alleles. Each dot represents one sample which is a pool of 10–15 flies. There were two different genetic backgrounds: round dots represent KK library RNAi lines and triangles GD library RNAi lines. The flies were reared at 18C where expression of the RNAi construct is inefficient and then transferred as adults to 25C. There was a significant heterogeneity among the KK library RNAi lines (*F* = 7.29, *P* = 3.6x10^-6^) but not among the GD lines.
(TIF)

S1 Table SNPs and indel found in the region (2L: 11094733–11102848) by comparing between resistant and susceptible flies. (XLS)

S2 Table Primers used for genotyping and sequencing. (XLS)
