# Correction: A Polymorphism in the Processing Body Component Ge-1 Controls Resistance to a Naturally Occurring Rhabdovirus in *Drosophila*

**DOI:** 10.1371/journal.ppat.1005730

**Published:** 2016-06-20

**Authors:** Chuan Cao, Michael M. Magwire, Florian Bayer, Francis M. Jiggins

The authors would like to correct four errors in the manuscript:

1. In Fig 5C, the relative viral titre bars are at the wrong height. In the figure, the *Ge-1*(S) ctrl (grey bar) and *Ge-1*(H) ctrl (grey bar) have higher relative viral titre than *Ge-1*(S) *DCP1* KD (red bar) and *Ge-1*(H) *DCP1* KD (blue bar). However, the *DCP1* KD should have higher viral titres than the controls in both S and H backgrounds. This mistake was made in R script when the figure was plotted. The text and legend are all correct and unaffected. Please see the corrected Fig 5 here.
2. The X labels of S2 Fig overlap. Label "*Edc3*" overlaps with each Ctrl1. This mistake is due to formatting errors. Unlike the error in Fig 5C this is just a cosmetic change. Please see the corrected S2 Fig here.
3. In the Results section, subsection "The resistant allele of *Ge-1* contains a 26 amino acid deletion", the authors would like to change the last sentence of the first paragraph of this section (Line 170–171) to: “We found that there was no consistent difference in gene expression in the resistant and susceptible lines at six days and twelve days after they had been injected with the virus (Fig 2D; day6: *F*_*1*,*76*_ = 0.0189,172 *p* = 0.891; day12: *F*_*1*,*97*_ = 6.109, *p* = 0.015)." This sentence describes the result more accurately.
4. There is an error in the Results section, subsection "Deleting the 26 amino acid region of *Ge-1* in transgenic flies makes them resistant to DMelSV". The word "higher" should be replaced with "lower" in the last sentence of the first paragraph of this section (Line 217). The corrected sentence is: "Similarly, viral titres were 512-fold higher in the transgenic line with the deletion than the three lines without the deletion (Fig 4B; *F*_*1*,*56*_ = 156.9, *P*<<0.001)." The results and conclusions are unaffected.

**Fig 5:**
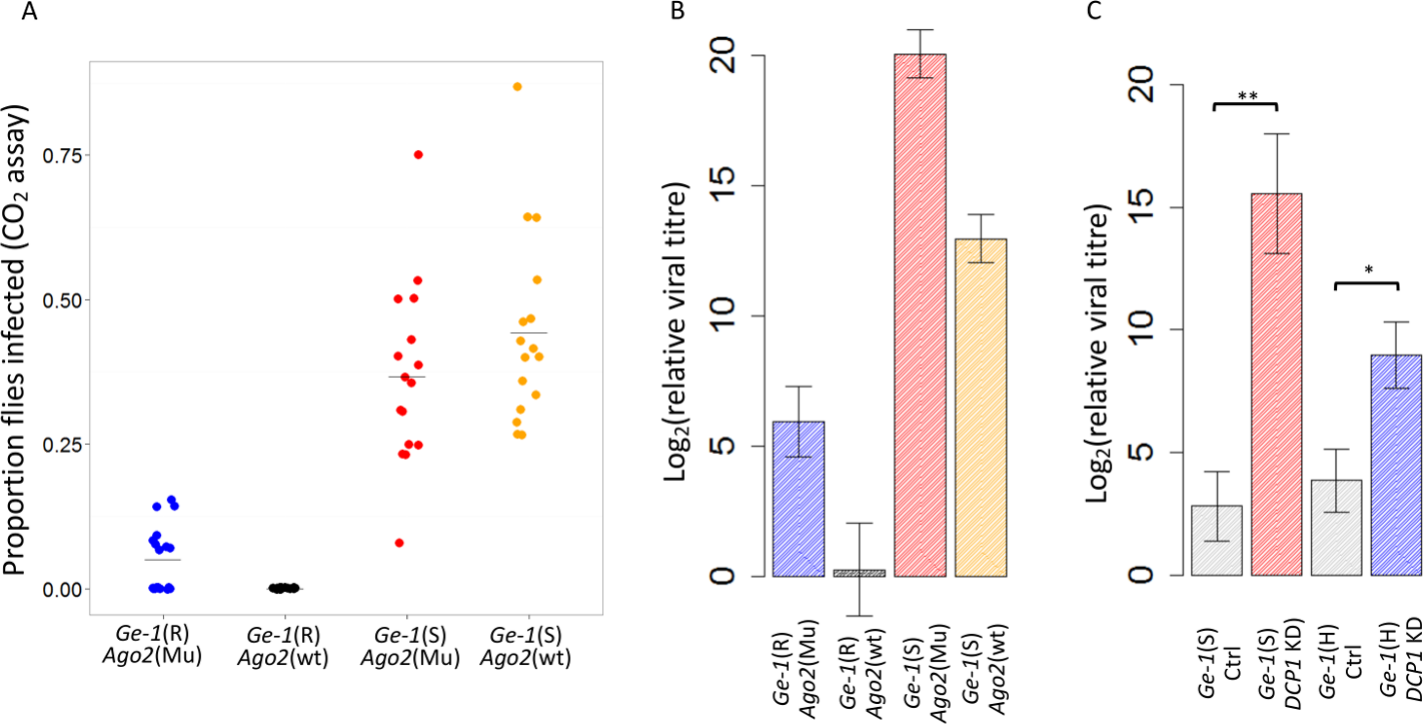
The effect of *Ago2* and *DCP1* on susceptibility to DMelSV. (A) and (B) are the effect of *Ge-1* on susceptibility in *Ago* ^*251B*^ mutant flies. Blue represents flies with the resistant *Ge-1* allele and *Ago2* mutant, black the resistant *Ge-1* allele and *Ago2* wild-type, red the *Ge-1* susceptible allele and *Ago2* mutant, and orange the susceptible *Ge-1* allele and *Ago2* wild-type. (A) CO_2_ sensitivity assay. (B) Relative viral titre estimated by quantitative PCR relative to *RpL32* was used as reference gene. (C) The effect of knocking down *DCP1* on viral titre in *Ge-1* susceptible backgrounds (red bar) and *Ge-1* heterozygous backgrounds (blue bar). Grey bars are controls for the RNAi. Error bars are standard errors.

## Supporting Information

S2 Fig RNAi knock-downs of P-body components. Eight genes encoding P-body components were knocked down by RNAi: *Edc3* (*CG6311*), *DCP2* (*CG6169*), *DCP1* (*CG11183*), *GW182* (*CG31992*), *pcm* (*CG3291*), *me31B* (*CG4916*), *Part-1* (*CG5208*) and *stau* (*CG5753*)). Left 10 boxes (red) are RNAi knock-downs in flies with *Ge-1* susceptible background. Right 10 boxes (blue) are RNAi knock-downs of flies that were heterozygous for the resistant and susceptible *Ge-1* alleles. Each dot represents one sample which is a pool of 10–15 flies. There were two different genetic backgrounds: round dots represent KK library RNAi lines and triangles GD library RNAi lines. The flies were reared at 18C where expression of the RNAi construct is inefficient and then transferred as adults to 25C. There was a significant heterogeneity among the KK library RNAi lines (*F* = 7.29, *P* = 3.6x10^-6^) but not among the GD lines.
(TIF)
