# Supplementary figures and images for: Correction: A Polymorphism in the Processing Body Component Ge-1 Controls Resistance to a Naturally Occurring Rhabdovirus in Drosophila
Source: PLoS Pathog. 2016 Jun 20;12(6):e1005730. doi: 10.1371/journal.ppat.1005730 (PMC4913908; doi:10.1371/journal.ppat.1005730)

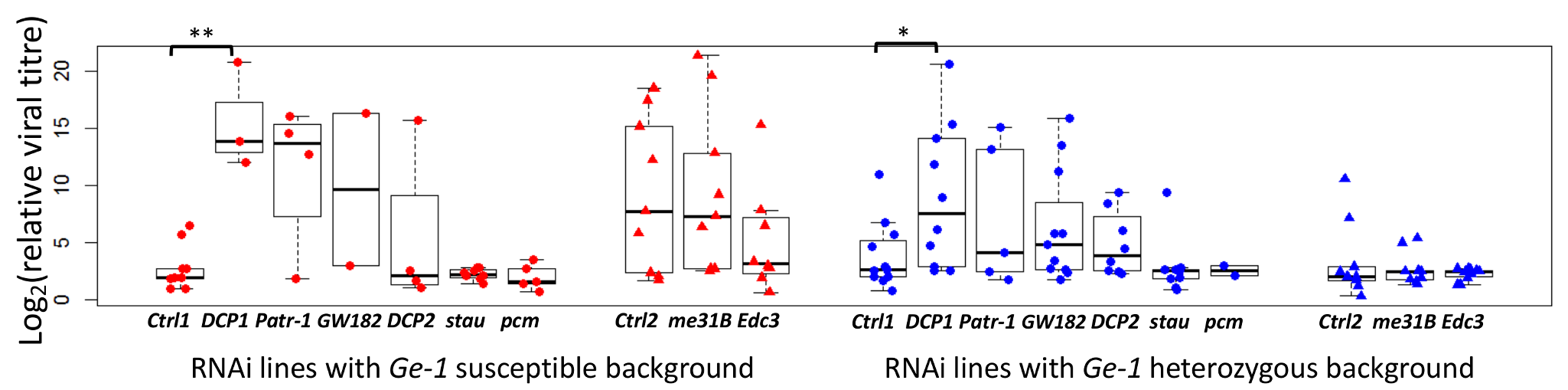

Supplement: S2 Fig — Eight genes encoding P-body components were knocked down by RNAi: Edc3 (CG6311), DCP2 (CG6169), DCP1 (CG11183), GW182 (CG31992), pcm (CG3291), me31B (CG4916), Part-1 (CG5208) and stau (CG5753)). Left 10 boxes (red) are RNAi knock-downs in flies with Ge-1 susceptible background. Right 10 boxes (blue) are RNAi knock-downs of flies that were heterozygous for the resistant and susceptible Ge-1 alleles. Each dot represents one sample which is a pool of 10–15 flies. There were two different genetic backgrounds: round dots represent KK library RNAi lines and triangles GD library RNAi lines. The flies were reared at 18C where expression of the RNAi construct is inefficient and then transferred as adults to 25C. There was a significant heterogeneity among the KK library RNAi lines (F = 7.29, P = 3.6x10^-6^) but not among the GD lines. (TIF) [file ppat.1005730.s001.tif]
